# β2-Microglobulin Elevates COL5A1 mRNA in the Subsynovial Connective Tissue of Patients Receiving Hemodialysis With Carpal Tunnel Syndrome

**DOI:** 10.7759/cureus.32423

**Published:** 2022-12-12

**Authors:** Kyoko Muneshige, Kenji Onuma, Koji Sukegawa, Yuya Otake, Gen Inoue, Masashi Takaso, Kentaro Uchida

**Affiliations:** 1 Department of Orthopedic Surgery, Kitasato University School of Medicine, Sagamihara, JPN; 2 Department of Orthopedic Surgery, Kitasato University, Sagamihara, JPN

**Keywords:** col5a1, subsynovial connective tissue, β2-microglobulin, hemodialysis, carpal tunnel syndrome

## Abstract

Background

Although carpal tunnel syndrome (CTS) is frequently observed in patients undergoing long-term hemodialysis (HD), exactly how CTS arises is unknown. Here, we examined levels of *COL5A1* in the subsynovial connective tissue (SSCT) of patients receiving HD and studied its potential regulation by β2-microglobulin (Β2-MG) in SSCT-derived cells (SSCTCs).

Methods

We extracted SSCT samples from 67 patients with CTS (49 non-HD and 18 HD) during carpal tunnel release. The samples were subjected to quantitative polymerase chain reaction (qPCR) to determine *COL5A1* expression. Further, to examine the potential regulation of *COL5A1* expression by Β2-MG, SSCTCs were stimulated in the absence (control) or presence of 10 µg/ml Β2-MG.

Results

The HD group showed significantly elevated *COL5A1* levels compared to the non-HD group (P=0.027). Moreover, treating SSCTCs with Β2-MG for 24 h increased the mRNA expression of *COL5A1* relative to control conditions (P=0.013).

Conclusions

Elevated *COL5A1* expression may form part of the mechanism underlying the development of CTS, and Β2-MG may play a role in promoting *COL5A1* expression in HD patients.

## Introduction

Carpal tunnel syndrome (CTS) is a frequently noted complication among patients receiving long-term hemodialysis (HD) [1-6]. In fact, the prevalence of CTS is reportedly linked to the duration of hemodialysis [2,3]. Despite this, exactly how CTS arises in these patients is unclear.

Histological investigations have noted fibrotic pathology of the subsynovial connective tissue (SSCT) in CTS patients [7]. Fibrosis changes the mechanical properties of SSCT in CTS patients as compared to healthy subjects [7-10]. Various organ and tissue fibrosis have also been observed in patients with HD [11,12]. In particular, type V collagen (COLV), which is classified as regulatory fibril-forming collagen [13], is overexpressed in the case of lung, liver, and skin fibrosis [13,14]. Further, variants within the three prime untranslated regions of the COL5A1 gene have been shown to be associated with idiopathic CTS [15]. However, the factors that alter COL5A1 expression in the SSCT of patients receiving HD with CTS have not been identified.

Insoluble fibrils of β2-microglobulin (Β2-MG) are involved in dialysis-related amyloidosis [16,17]. Β2-MG is also associated with inflammation and fibrosis in the liver, heart, and kidney [18-27]. Notably, amyloid deposits have been noted in the synovium of patients undergoing HD with CTS [28-30]. However, the role of COL5A1 in SSCT has not been studied.

p38 mitogen-activated protein kinase (p38 MAPK) mediates a pivotal intracellular signal transduction pathway, and activation of p38MAPK is reported to be involved in several physiological responses, including inflammation, stress responses, and apoptosis [31]. In addition, p38 MAPK has been demonstrated to contribute to the pathogenesis of fibrotic conditions [32-35]. A previous study suggested that B2-MG may trigger fibrosis mechanisms through the p38 pathway in kidney diseases [36]. However, exactly how the p38 pathway contributes to COL5A1 expression in SSCTs remains unclear.

To identify a possible cause of CTS in HD patients, we examined COL5A1 expression in patients’ SSCT. We also examined the potential regulation of COL5A1 by Β2-MG in SSCT-derived cells (SSCTCs) to determine a potential pathway through which to reduce COL5A1 expression.

## Materials and methods

Patients

This study was approved by the Ethics Committee at the Clinical Research Review Board of Kitasato Institute (reference no: B13-113) and abides by the 1964 Helsinki Declaration and its later amendments or comparable ethical standards. All participants provided written informed consent.

To study COL5A1 expression in the SSCT of HD and non-HD patients, we extracted SSCT from patients with CTS during carpal tunnel release (CTR). Of the 124 patients who received CTR, we excluded 36 whose body mass index (BMI) was >25 kg/m^2^, which was used to indicate overweight or obesity because BMI is a risk factor for CTS [37]. All patients were subjected to diagnostic neurophysiological tests that included electromyography and nerve conduction tests performed according to the American Association of Electrodiagnostic Medicine standards to confirm their CTS diagnosis [38]. Subsequently, we additionally excluded patients with a history of traumatic injuries, peripheral nerve disease, sarcoidosis, flexor tendinitis, osteoarthritis, rheumatoid arthritis, and thyroid disease according to information on their medical charts. Ultimately, samples from 67 patients with CTS (49 non-HD and 18 HD) were included for analysis. Of these, samples from seven male and three female non-HD patients with an average age of 65.1±9.0 years (range 52 to 78 years) were used to examine the pathological role of Β2-MG in patients with CTS. Further, samples from six male and four female non-HD patients with an average age of 70.4±12.5 years (range 54 to 90 years) were used to examine the role of Β2-MG in patients with CTS.

SSCTCs culture

To isolate SSCTCs, SSCT specimens were treated with clostridium histolyticum-derived 0.1% type I collagenase for 24 h at 37°C. SSCTCs were cultured for two weeks in α-MEM supplemented with 10% fetal bovine serum (FBS) and 10 ng/ml fibroblast growth factor-2 (FGF2). After the two-week incubation, we confirmed that SSCTCs were negative for the hematopoietic cell marker, CD45, and positive for the fibroblast marker, CD90, using flow cytometry. To prevent any potential effects of 10% FBS and FGF2 on COL5A1 expression, SSCTCs were washed three times with PBS before replacing the medium with α-MEM containing 0.5% FBS. Three hours later, SSCTCs were exposed to α-MEM (control) with 0.5% FBS or 10 µg/ml Β2-MG. To evaluate the effect of p38 inhibition on COL5A1 expression, SSCTCs were stimulated with B2-MG in the absence (control) or presence of 10 µM SB203580 (a p38 inhibitor).

Quantitative polymerase chain reaction (PCR)

To extract total RNA from SSCT, SSCT samples were homogenized in TRIzol reagent using a homogenizer and then treated with Direct-zolTM RNA Micro Prep (Zymo Research, Irvine, CA). cDNA synthesis was subsequently performed on the purified total RNA (260/280 = 1.8-2.0) using reverse transcriptase (SuperScript III kit, Life Technologies, ThermoFisher, Waltham, MA). Primers used in the PCR reactions are provided in Table 1. To determine COL5A1 mRNA expression, we subsequently performed quantitative PCR analysis using SYBR Green (Bio-Rad, CA, USA). COL5A1 expression levels were normalized to that of glyceraldehyde 3-phosphate dehydrogenase (GAPDH) and analyzed using the 2−ΔΔCt method.

**Table 1:** Primers used in the study

Gene	Direction	Primer Sequence (5¢–3¢)	Product Size (bp)
COL5A1	F	AAGCGTGGGAAACTGCTCTC	114
	R	GTGGTAGGTGACGTTCTGGT	
GAPDH	F	TGTTGCCATCAATGACCCCTT	202
	R	CTCCACGACGTACTCAGCG	

Statistical analysis

The Shapiro-Wilk test was first used to determine whether our data were normally distributed. As our data were not normally distributed, we used the Mann-Whitney U test for the main analysis. P values less than 0.05 were used to indicate statistical significance in all tests. Data were analyzed using SPSS software v.28.0 (IBM Corp., Armonk, NY).

## Results

Patient clinical data

The clinical data of patients in the HD and non-HD groups are summarized in Table 2. Although gender was significantly different between the HD and non-HD groups (P=0.050), no significant differences were noted in age or BMI (age, P=0.791; BMI, P=0.354).

**Table 2:** Patients’ demographic data BMI, body mass index

	Hemodialysis	Non-hemodialysis	P value
Age	69.3±6.5	70.0±12.2	P=0.791
Sex (Female/Male)	7/11	33/16	P=0.050
BMI	20.9±1.9	21.5±2.3	P=0.354

COL5A1 expression in SSCT of HD and non-HD patients

**Figure 1:**
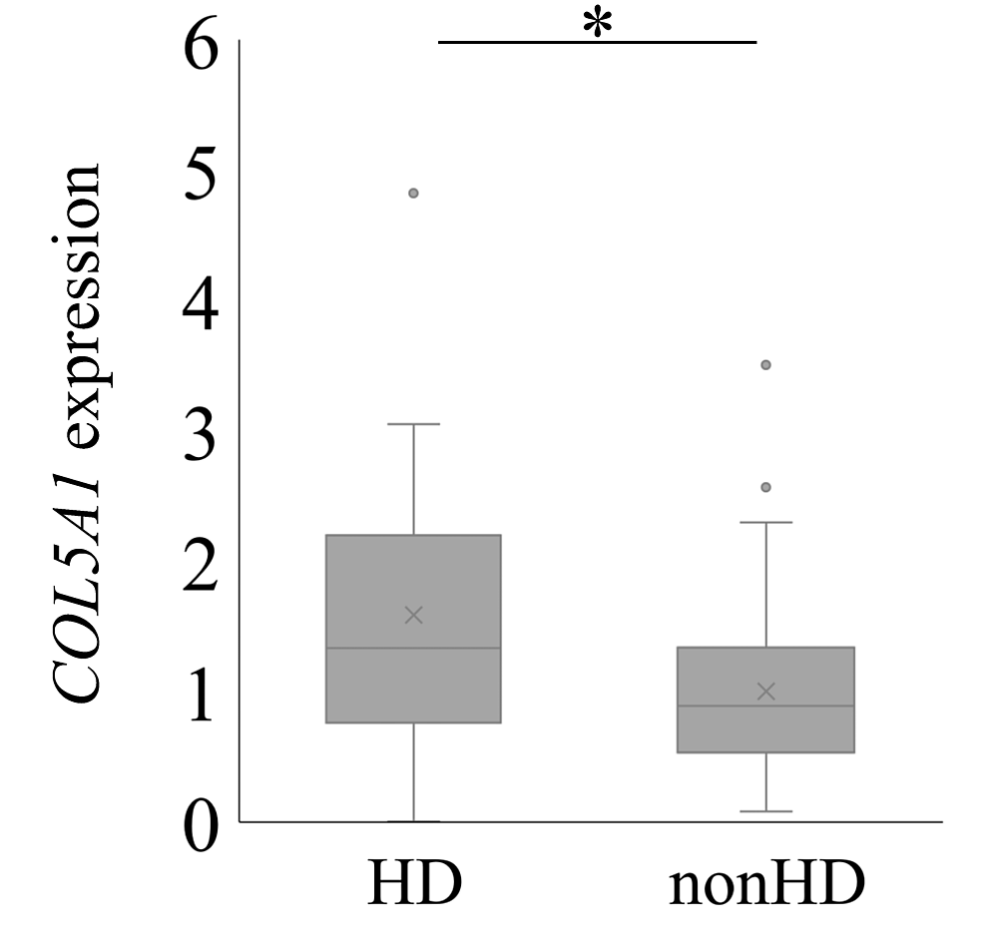
Expression of COL5A1 in the subsynovial connective tissue of hemodialysis (HD) and non-hemodialysis (non-HD) patients with carpal tunnel syndrome COL5A1 *p<0.05

To examine the fibrotic condition of SSCT, we studied COL5A1 expression using quantitative PCR. The HD group showed significantly elevated COL5A1 levels relative to the non-HD group (P=0.027; Figure 1).

Regulation of COL5A1 expression by B2-MG in SSCTCs

**Figure 2:**
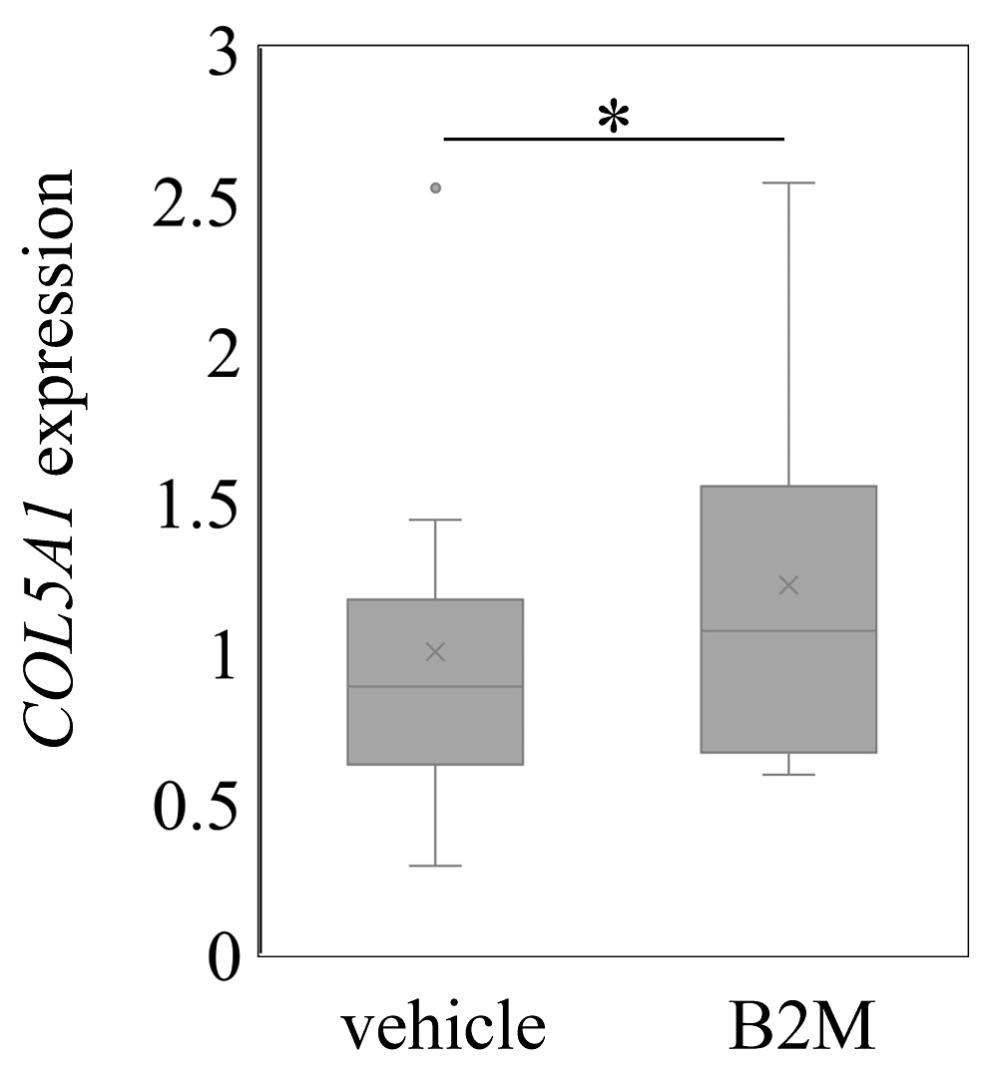
Expression of COL5A1 in subsynovial connective tissue cells following exposure to Β2-MG COL5A1 levels in subsynovial connective tissue cells following exposure to 0 (control) or 10 μg/ml Β2-MG. *p<0.05

Exposing SSCTCs to Β2-MG for 24 h elevated COL5A1 expression levels compared to control conditions (P=0.013; Figure 2).

Regulation of COL5A1 by p38 in SSCTCs

**Figure 3:**
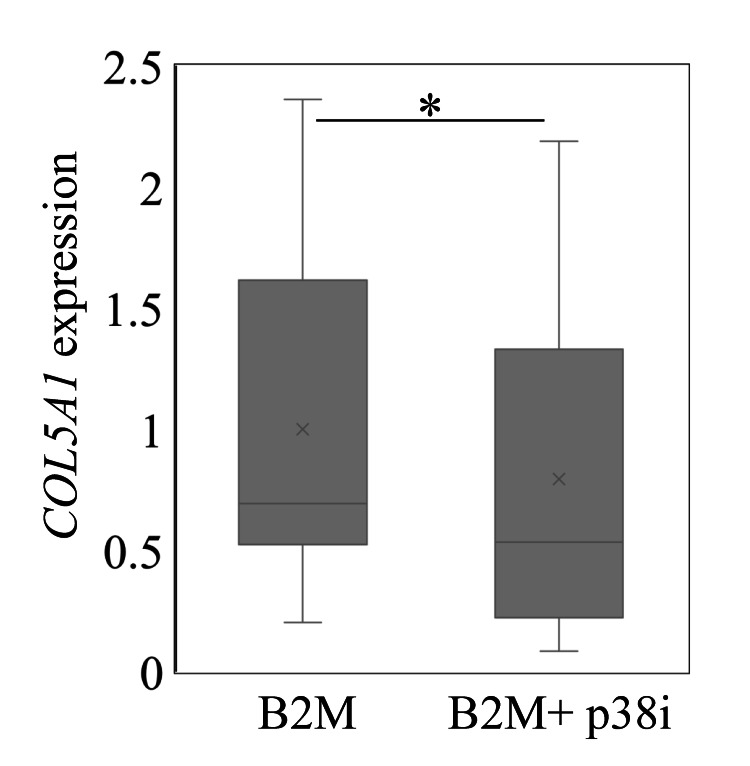
Effect of a p38 MAPK inhibitor on COL5A1 expression in subsynovial connective tissue cells following exposure to Β2-MG COL5A1 levels in subsynovial connective tissue cells following exposure to 10 μg/ml Β2-MG or B2-MG with a p38 inhibitor (p38i). *p<0.05

Treatment of SSCTCs with a p38 inhibitor significantly reduced COL5A1 expression compared to treatment with B2M alone (P=0.025; Figure 3).

## Discussion

We showed that HD patients had higher levels of COL5A1 than non-HD patients with CTS. Moreover, exposing SSCTCs to Β2-MG increased COL5A1 expression, suggesting that Β2-MG could play a role in the development of CTS in HD patients.

Previous studies have reported increased COL5A1 levels in fibrotic disorders. For example, elevated mRNA expression of COL5A1 and impaired ColV deposition are associated with fibrosis and worsening function of pulmonary tissues in systemic sclerosis [39]. Here, we found that HD patients had higher COL5A1 expression than non-HD patients with CTS. Previous studies have also reported increased COL1A1 and COL3A1 expression in the SSCT of CTS patients compared to healthy controls. Further, increased COLV relative to COLI/III has been noted in the human aorta in cases of atherosclerosis [13]. Therefore, elevated COL5A1 in SSCT may be a risk factor for CTS in patients undergoing HD.

Β2-MG is a small membranous protein that binds to MHC class I on nucleated cells [40]. B2-MG is typically removed by proximal tubular reabsorption and glomerular filtration [40]. However, patients with chronic kidney disease have markedly decreased B2-MG catabolism. Elevated plasma Β2-MG concentrations cause deposits of Β2-MG to accumulate in tissues. A previous study showed that Β2-MG treatment of chondrocyte cells taken from knee osteoarthritis patients promoted COL3A1 expression [41]. Similarly, we showed that treatment of SSCTCs with Β2-MG stimulated COL5A1 expression. Thus, the accumulation of Β2-MG in the SSCTCs of HD patients could induce CTS by enhancing COL5A1 expression.

Previous studies have reported that p38 MAPK regulates collagen gene expression. p38 MAPK was shown to regulate Col1a1 expression in a rat hepatic stellate cell line [42]. Further, SB203580 inhibited TGF-β-mediated COL1A1 expression in human bronchial fibroblasts [43]. Moreover, IL-17-mediated COL5A1 expression was blocked by inhibitors of the p38 MAPK signaling pathway in human small airway epithelial cells [44]. Consistent with previous findings, we showed that a p38 inhibitor reduced COL5A1 expression in SSCTCs. Inhibition of the p38 pathway may thus be a pharmacological strategy for treating fibrosis of SSCTs associated with HD.

Several limitations of the present study warrant mention. First, the findings of this study may not be generalizable to patients outside Asia, who tend to have higher BMI than those in Asian countries. Second, the small sample size may have led to type II errors. Third, we did not determine patients’ average duration of dialysis. Fourth, we did not examine a control population. The inclusion of a non-CTS population is needed to confirm whether COL5A1 expression is increased in CTS with HD. Fifth, we only assessed COL5A1 mRNA expression in SSCT. Further investigation, such as a protein profiling study using western blotting, is needed to complement our gene expression results. Finally, the relationship between COL5A1 expression and CTS pathology remains to be determined.

## Conclusions

We examined levels of COL5A1 in the SSCT of patients receiving HD and studied its potential regulation by Β2-MG in SSCTCs. We found that COL5A1 was elevated in the SSCT of CTS patients with HD. Further, COL5A1 increased following B2B treatment and decreased following exposure to a p38 MAPK inhibitor. Elevated COL5A1 expression may thus form part of the mechanism underlying the development of CTS and Β2-MG may play a role in promoting COL5A1 expression in HD patients. Thus, COL5A1 and Β2-MG may be important therapeutic targets for the treatment of CTS associated with HD.
